# Primary Infection May Be an Underlying Factor Contributing to Lethal Hemorrhagic Disease Caused by Elephant Endotheliotropic Herpesvirus 3 in African Elephants (*Loxodonta africana*)

**DOI:** 10.1128/Spectrum.00983-21

**Published:** 2021-10-20

**Authors:** Taylor Pursell, Jennifer L. Spencer Clinton, Jie Tan, Rongsheng Peng, Xiang Qin, Harshavardhan Doddapaneni, Vipin Menon, Zeineen Momin, Kavya Kottapalli, Lauren Howard, Erin Latimer, Sarah Heaggans, Gary S. Hayward, Paul D. Ling

**Affiliations:** a Department of Molecular Virology and Microbiology, Baylor College of Medicine, Houston, Texas, USA; b Human Genome Sequencing Center, Baylor College of Medicine, Houston, Texas, USA; c San Diego Zoo Safari Park, San Diego Zoo Wildlife Alliance, Escondido, California, USA; d Wildlife Health Sciences, Smithsonian’s National Zoo, Washington, DC, USA; e Viral Oncology Program, Johns Hopkins School of Medicine, Baltimore, Maryland, USA; Barnard College, Columbia University

**Keywords:** elephant, herpesvirus, serology

## Abstract

Distinct but related species of elephant endotheliotropic herpesviruses (EEHVs) circulate within Asian and African elephant populations. Primary infection with EEHVs endemic among Asian elephants can cause clinical illness and lethal EEHV hemorrhagic disease (EEHV-HD). The degree to which this occurs among African elephants has not been fully established. Recent cases of EEHV-HD caused by the EEHV3 species in African elephants housed in North American zoos has heightened concern about the susceptibility of this elephant species to EEHV-HD. In this study, we utilize the luciferase immunoprecipitation system (LIPS) to generate a serological assay specific for EEHV3 in African elephants by detecting antibodies against the EEHV3 E34 protein. The results showed that the majority of tested elephants from four separate and genetically unrelated herds, including five elephants that survived clinical illness associated with EEHV3, were positive for prior infection with EEHV3. However, African elephants who succumbed to EEHV3-HD were seronegative for EEHV3 prior to lethal infection. This supports the hypothesis that fatal EEHV-HD caused by EEHV3 is associated with primary infection rather than reactivation of latent virus. Lastly, we observed that African elephants, like Asian elephants, acquire abundant anti-EEHV antibodies prenatally and that anti-EEHV3 specific antibodies were either never detected or declined to undetectable levels in those animals that died from lethal disease following EEHV3 infection.

**IMPORTANCE** Prior to 2019, only five cases of clinical disease from EEHV infection among African elephants had been documented. Since 2019, there have been at least seven EEHV-HD cases in North American zoos, resulting in three fatalities, all associated with EEHV3. Evidence is accumulating to suggest that EEHV-associated clinical illness and death among Asian elephants is due to primary infection and may be associated with waning anti-EEHV antibody levels in young elephants. The development of the EEHV3 serological test described in this study enabled us to confirm that similar dynamics may be contributing to EEHV-HD in African elephants. The ability to screen for EEHV immune status in African elephant calves will have a major impact on managing captive African elephant herds and will provide new tools for investigating and understanding EEHV in wild populations.

## INTRODUCTION

Elephant endotheliotropic herpesvirus (EEHV) can cause lethal EEHV hemorrhagic disease (EEHV-HD) in both captive and wild elephants (1, 2). Although morbidity and death have been documented in both Asian elephants (Elephas maximus) and African elephants (Loxodonta africana) (3, 4), the majority of severe clinical illnesses and fatalities are seen in juvenile Asian elephants (1). Recent evidence suggests that clinical illness and lethal EEHV-HD in Asian elephants are caused by primary infection rather than reactivation of latent virus (5). Due to the low incidence of documented clinical disease caused by EEHV in African elephants, the biology of these viruses, compared to those endemic among Asian elephants, is lesser known. Since 2019, however, at least seven cases of EEHV-HD in African elephants, resulting in three fatalities, have occurred (6), suggesting possible increasing significance of EEHV in African elephants. These cases have also revealed gaps in knowledge and the need for diagnostic tools to study and to monitor EEHV in this elephant species.

Multiple EEHV species have now been identified, and the genomic sequences for prototypical representatives of each species in Asian elephants have been determined (7–10). Evidence suggests that EEHV2, EEHV3, EEHV6, and EEHV7 circulate naturally among African Savannah elephants, while EEHV1, EEHV4, and EEHV5 are endemic among Asian elephants (1). These viruses can be further subdivided, based on their genomic composition, into either the AT-rich group, containing EEHV1, EEHV2, EEHV5, and EEHV6, or the GC-rich group, containing EEHV3, EEHV4, and EEHV7 (1, 4, 7, 8, 11–14). The GC-rich branch has a 15% increase in GC content concentrated at the wobble codon within the majority of codon regions. Regardless of host species, viruses within the GC-rich group or the AT-rich group share significant amino acid identity (i.e., 65% to 80%) with each other in their core proteins but retain only 30% to 50% amino acid identity over one-half or less of their length in the novel *Proboscivirus* genus-specific proteins. The majority of EEHV-HD cases in captive African elephants have been caused by a single GC-rich species, EEHV3 (3, 6). Recent sequencing studies from our laboratories have identified at least two distinct chimeric subspecies of EEHV3, i.e., EEHV3A and EEHV3B, which diverge by at least 9% on average across their genomes (15) (G. S. Hayward and P. D. Ling, unpublished data). The extent to which these virus subspecies circulate within captive and wild elephants remains unclear. In addition, it remains unknown whether infection with one subspecies can protect against infection or disease from the other.

Previous work with Asian elephants provided evidence that clinical illness and death are associated with primary infection and may correlate with waning anti-EEHV antibody levels in young elephants (5). Therefore, serological tests to assess the serostatus of vulnerable populations could play a key role in the monitoring of the risk of EEHV-HD in these animals. With that aim, a serological assay for EEHV, utilizing the luciferase immunoprecipitation system (LIPS) (5, 16) and a panel of EEHV recombinant biomarkers, has been validated in Asian elephants. The goal of this study was to identify and to validate an immunoreactive biomarker for the detection of pan-EEHV antibodies, as well as EEHV3-specific antibodies, in African elephants, utilizing the previously published and validated LIPS assay platform (5, 16). By leveraging the divergence of the GC-rich branch from the AT-rich branch of EEHVs, we were able to select and to validate a potential biomarker to detect specific antibodies for the GC-rich EEHV3 in African elephants.

## RESULTS

### The LIPS assay is sensitive and specific for detecting EEHV U39 and E34 antibodies.

To validate the LIPS assay for use in African elephants, we assessed antibody responses in two distinct groups, i.e., (i) a family herd of 11 elephants in which at least 5 herdmates have demonstrated EEHV3 shedding or viremia, suggesting that most or all herd members have been exposed and are chronically infected (Table 1), and (ii) elephants that died from EEHV3-associated HD (EEHV-HD group I) (6) (Table 2). Drawing on studies in Asian elephants (5), we hypothesized that fatal EEHV3-HD was due to a primary infection and thus these animals should lack immunoreactivity against EEHV3-specific proteins. We tested serum from the aforementioned elephant cohorts for immunoreactivity against two proteins, namely, U39 (glycoprotein B [gB]) and E34 (open reading frame C [ORF-C]), from a representative EEHV3A strain. Similar to Asian elephant EEHVs, the U39 protein is generally well conserved across EEHV2, EEHV3, and EEHV6 (Table 3). It was anticipated that immunoreactivity to U39 would be detected in most elephants, as long as they had been infected previously with at least one EEHV species. E34 was chosen as a putative biomarker to detect specific responses to EEHV3 versus the other EEHV species endemic among African elephants because it diverges significantly from that in the AT-rich branch species (i.e., EEHV2 and EEHV6) (Table 3) and resides in a genomic location that is relatively invariant among EEHV strains within each species (1, 8, 13, 14). Specifically, only 404 amino acid residues are conserved between EEHV3A (2,059 amino acids) and EEHV2 (1,858 amino acids), averaging 19% identity across each of their primary sequences, with most of the homology residing in the amino-terminal end. In contrast, 1,550 amino acid residues are conserved between EEHV3A and EEHV3B E34 proteins, averaging 77% identity across their primary amino acid sequences (Table 3). EEHV3 and EEHV2 E34 proteins are 21% and 32% identical, respectively, to EEHV6 (Table 3). Therefore, it was predicted that the EEHV3 E34 protein could be a specific biomarker to distinguish antibodies generated from infection with EEHV3 versus those in the AT-rich branch, although it’s unlikely there is sufficient divergence (77% identical) to distinguish between EEHV3A and EEHV3B infections.

**TABLE 1:** Summary of data for African elephants with evidence of prior-EEHV exposure^*a*^

Elephant	Sex^*b*^	Date of birth (yr or mo/day/yr)	Origin (birth)	Sample date (mo/day/yr)^*c*^
SDZSP-1	M	1989	Wild	7/8/19
SDZSP-2	M	3/13/2009	Captive	6/19/19
SDZSP-3	M	2/14/2010	Captive	6/19/19
SDZSP-4	M	9/26/2011	Captive	8/15/19
SDZSP-5	M	5/13/2010	Captive	4/1/19
SDZSP-6	F	8/28/2012	Captive	8/15/19
SDZSP-7	F	9/11/2006	Captive	8/15/19
SDZSP-8	M	4/12/2010	Captive	4/6/18
SDZSP-9	F	1990	Wild	7/11/19
SDZSP-10	F	1990	Wild	8/6/19
SDZSP-11	F	9/19/2007	Captive	8/14/19

a Positive EEHV3 quantitative PCR results from trunk wash, whole-blood, or fecal samples from at least 5 members of the group.

b M, male; F, female.

c Date of serum sample collection.

**TABLE 2:** Summary of data for eight EEHV3-associated HD cases in African elephants evaluated in these studies

Elephant	Herd	Sex^*a*^	Origin (birth)	Date of birth (yr or mo/day/yr)	EEHV species	Case no.	Outcome	EEHV-HD group	Sample date (mo/day/yr)^*b*^	Date of illness onset (mo/day/yr)
FCZ-1	B	F	Captive	12/8/2007	3B	NAP86	Fatal	I	1/24/19	2/17/19
IDZ-1	A	F	Captive	6/28/2012	3A	NAP97	Fatal	I	9/11/18	3/17/19
IDZ-2	A	F	Captive	8/1/2019	3A	NAP98	Fatal	I	9/11/18	3/23/19
MZiB-1	C	M	Captive	3/19/2008	3B	NAP62	Survived	II	9/5/12	2/26/13
FCZ-2	B	F	Wild	2009^*c*^	3B	NAP99	Survived	II	2/25/19	4/29/19
IDZ-3	A	M	Captive	7/31/2005	3A	NAP100	Survived	II	4/29/19	5/6/19
IDZ-4	A	F	Captive	8/31/2006	3A	NAP101	Survived	II	5/14/19	5/18/19
IDZ-5	A	F	Wild	1982	3A	NAP102	Survived	II	6/13/19	7/18/19

a F, female; M, male.

b Most recent available serum sample prior to detection of illness.

c Estimated.

**TABLE 3:** Protein relatedness across EEHV species endemic in African elephants

Protein	Amino acid sequence identity (%)					
	EEHV2 vs EEHV6	EEHV2 vs EEHV3A	EEHV2 vs EEHV3B	EEHV3A vs EEHV3B	EEHV3A vs EEHV6	EEHV3B vs EEHV6
U39 (gB)	82	66	66	94	63	64
E34 (ORF-C)	32	20	19	77	21	21

**FIG 1:**
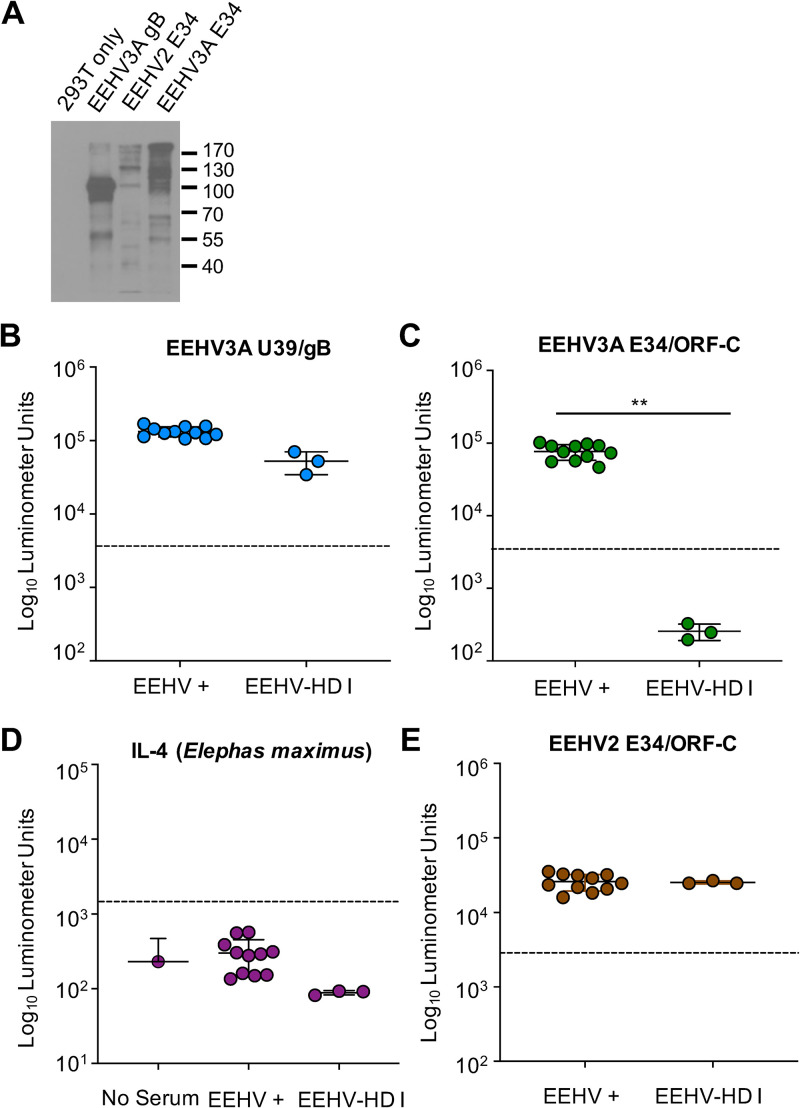
Detection of anti-EEHV antibodies by LIPS assays. (A) Immunoblot of EEHV-GLuc fusion proteins detected with anti-GLuc antibodies. Proteins are labeled at the top of the blot, with the sizes of protein markers indicated to the right. (B and C) Detection of anti-EEHV3 U39/gB (B) and EEHV3A E34/OF-C (C) antibodies by LIPS assays. Each symbol represents a sample from an animal with evidence of prior EEHV infection(s) (EEHV+) and those that died from EEHV3-HD (EEHV-HD I). Antibody levels are expressed in RLUs and plotted on a log_10_ scale. Mean ± SD values for each cohort (EEHV-positive or EEHV-HD I) are shown, with each symbol representing the mean result for one elephant at a single time point with four replicates from two independent experiments. **, statistically significant difference (*P < *0.005) between EEHV-positive and EEHV-HD I groups, as determined by the Mann-Whitney *U* test on log-transformed values. The dashed lines indicate the cutoff levels for determining the sensitivity and specificity for each viral antigen, which were derived from the mean antibody titers of seronegative serum samples or no-serum controls plus 5 SDs. (D) An Asian elephant IL-4-GLuc fusion protein was used to measure antibodies to an elephant protein in both seropositive and seronegative elephants, and the data were compared with no-serum controls. (E) Detection of anti-EEHV2 E34 antibodies by LIPS as described for panels A and B.

The EEHV3A U39, EEHV3A E34, and EEHV2 E34 proteins were expressed as fusions with the *Gaussia* luciferase (GLuc) protein for the LIPS assay, as described previously (5, 17) (Fig. 1A). Initially, we tested random serum samples collected from the EEHV-positive group between April 2018 and August 2019 and from EEHV-HD group I at available time points nearest the onset of clinical illness. This included serum samples collected 6 months prior to detection of clinical illness (September 2018) for IDZ-1 and IDZ-2 and about 3 weeks prior (January 2019) for FCZ-1 (Table 2). We detected positive responses to our pan-EEHV marker, U39, in the EEHV3-positive group, including the three lethal EEHV-HD cases (EEHV-HD group I) (Fig. 1B). This suggests that all of the elephants tested had been previously infected by at least one EEHV, including those elephants that died from EEHV-HD. The animals in the EEHV-positive group were also immunoreactive to the EEHV3-specific biomarker, E34 (Fig. 1C). In contrast, the most recently collected serum samples prior to EEHV3-associated HD for the EEHV-HD group I elephants showed no immunoreactivity to EEHV3 E34 (Fig. 1C). Additionally, there was no difference between the EEHV-HD group I samples and no-serum controls (*P* = 0.2) (data not shown). To further validate the assay, we screened the serum samples from the EEHV-positive and EEHV-HD group I cohorts against the E. maximus interleukin 4 (IL-4) (100% identical to Loxodonta africana IL-4)-GLuc fusion protein in the LIPS assay, which yielded results similar to those for the no-serum controls (Fig. 1D). These data suggest that we can utilize no-serum controls as negative controls for comparison, to conserve valuable serum samples from seronegative animals, which are limited in quantity. To identify a possible explanation for the reactivity of the EEHV-HD group I samples to our pan-EEHV marker, EEHV3A U39, we tested the serum samples for immunoreactivity to EEHV2 E34. Both EEHV-positive and EEHV-HD group I elephants were immunoreactive to EEHV2 E34, indicating that all of these elephants had been infected previously with EEHV2 or possibly EEHV6 (due to potential cross-reactivity), or both (Fig. 1E). This result also indicates that the EEHV-HD group I animals were capable of generating antibody responses to an E34 protein from a related virus, further supporting the observation that lack of reactivity to EEHV3 E34 was because those animals were naive regarding infection with that virus species at times relatively close to the onset of lethal infection with EEHV3.

### African elephants that survived clinical illness from EEHV3 were seropositive for EEHV3 prior to illness.

**FIG 2:**
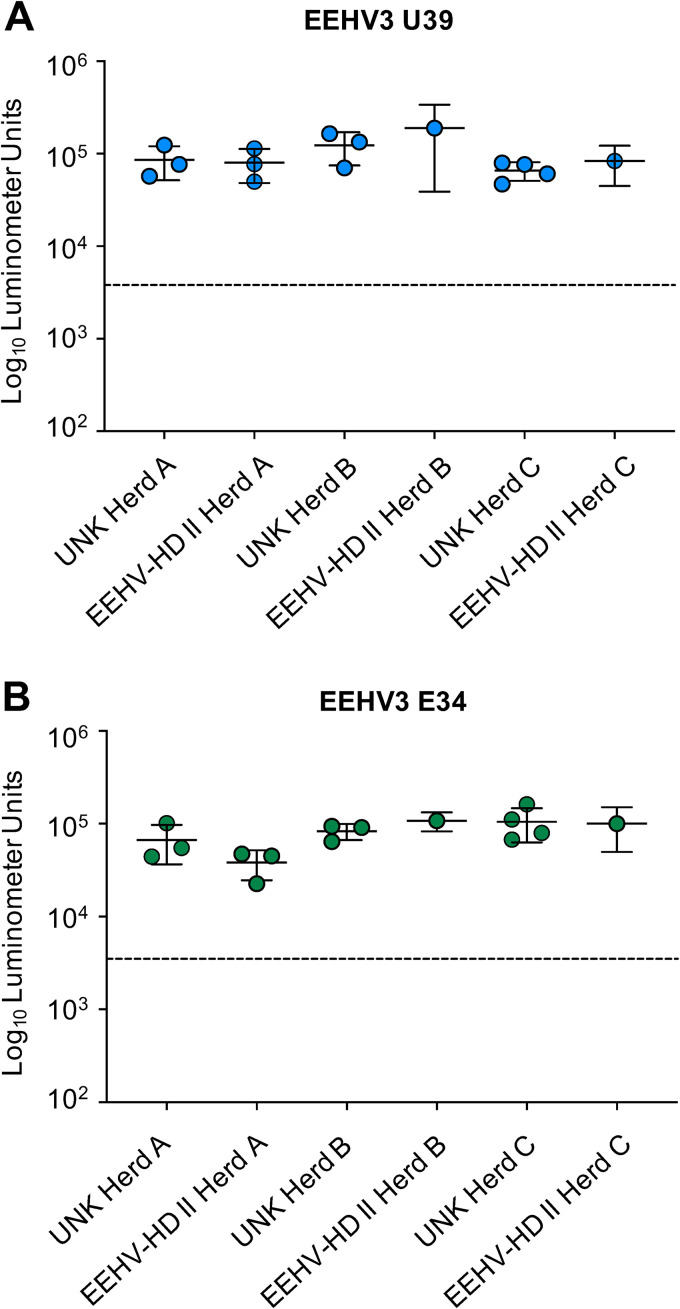
Screening for anti-EEHV antibodies in an uncharacterized cohort of adult elephants and in elephants that survived EEHV-HD, with detection of anti-EEHV3A U39 (A) and EEHV3A E34 (B) antibodies by LIPS assays. Each symbol represents a sample from an animal either with unknown EEHV history (UNK) or with a history of surviving EEHV3-HD (EEHV-HD II) from one of three herds (A, B, or C). Antibody levels are expressed in RLUs and plotted on a log_10_ scale. Mean ± SD values for each cohort (unknown-history, EEHV-positive, EEHV-HD I, and EEHV-HD II groups) are shown, with each symbol representing the mean result for one elephant at a single time point with four replicates from two independent experiments. **, statistically significant difference (*P < *0.005) between unknown-history, EEHV-positive, EEHV-HD I, and EEHV-HD II groups, as determined by the Mann-Whitney *U* test on log-transformed values. The dashed lines indicate the cutoff levels for determining the sensitivity and specificity for each viral antigen, which were derived from the mean antibody titers of seronegative serum samples or no-serum controls plus 5 SDs.

Having established assays to detect anti-pan-EEHV and anti-EEHV3 specific antibodies in African elephant sera, we used them to interrogate two additional groups of elephants, i.e., (i) adult elephants from three different herds with unknown EEHV history for some of the individuals (Table 4) and (ii) five elephants within each of the same herds that survived EEHV3-HD (EEHV-HD group II) (Table 2). For EEHV-HD group II elephants, serum samples ranging from 5 months to 4 days prior to the onset of clinical illness from EEHV3 were tested (Table 2). The elephants from both groups were immunoreactive to U39 at levels similar to those of the EEHV-positive control group (Fig. 2A). Thus, all of the adult African elephants tested, as well as elephants that suffered EEHV3-HD, had been infected with at least one EEHV previously, regardless of whether they survived a known EEHV-HD infection. When screened for EEHV3-specific antibodies using EEHV3A E34, the elephants with unknown EEHV history showed positive responses similar to those of the EEHV-positive control group (Fig. 2B). Interestingly, the EEHV-HD group II animals were also immunoreactive to EEHV3A E34 (Fig. 2B). Of those individuals, two animals (MZiB-1 and FCZ-2) experienced EEHV-HD caused by EEHV3B, while IDZ-3, IDZ-4, and IDZ-5 EEHV-HD cases were associated with EEHV3A (Table 2). Because of the similarity between EEHV3A and EEHV3B E34 proteins, it is unlikely that this biomarker will be sufficient to distinguish antibody responses generated after infection with these individual virus species. In Asian elephants, it has been documented that EEHV-HD caused by EEHV1A is not always sufficient to prevent illness caused by a related species, EEHV1B, and infection with EEHV1B is not sufficient to provide protection from illness caused by EEHV1A (18–20). EEHV1A and EEHV1B have an average divergence across their genomes of approximately 4.5%. However, the average divergence between EEHV3A and EEHV3B is somewhat greater and approaches 9% (G. S. Hayward and P. D. Ling, unpublished data). Additional biomarkers specific for EEHV3A or EEHV3B will be required to determine whether elephants that develop clinical illness from one EEHV3 subspecies generate some level of protective immunity against the other.

**TABLE 4:** Summary of data for African elephants from different herds evaluated in these studies

Elephant	Herd	Sex^*a*^	Date of birth (yr or mo/day/yr)	Origin (birth)	Sample date (mo/day/yr)^*b*^
IDZ-6	A	F	1968	Wild	9/11/2018
IDZ-7	A	F	1976	Wild	9/11/2018
IDZ-8	A	F	1976	Wild	9/11/2018
FCZ-3	B	F	1987	Wild	12/7/17
FCZ-4	B	F	1994	Wild	5/27/19
FCZ-5	B	M	2/23/2004	Captive	5/3/19
MZiB-2	C	F	1975	Wild	7/6/20
MZiB-3	C	F	1983	Wild	7/6/20
MZiB-4	C	M	1983	Wild	8/23/11
MZiB-5	C	F	1977	Wild	3/5/14

a F, female; M, male.

b Date of serum sample collection.

### Maternal anti-EEHV3 antibodies are prenatally transferred, and their titers can decline in some juvenile African elephants.

**FIG 3:**
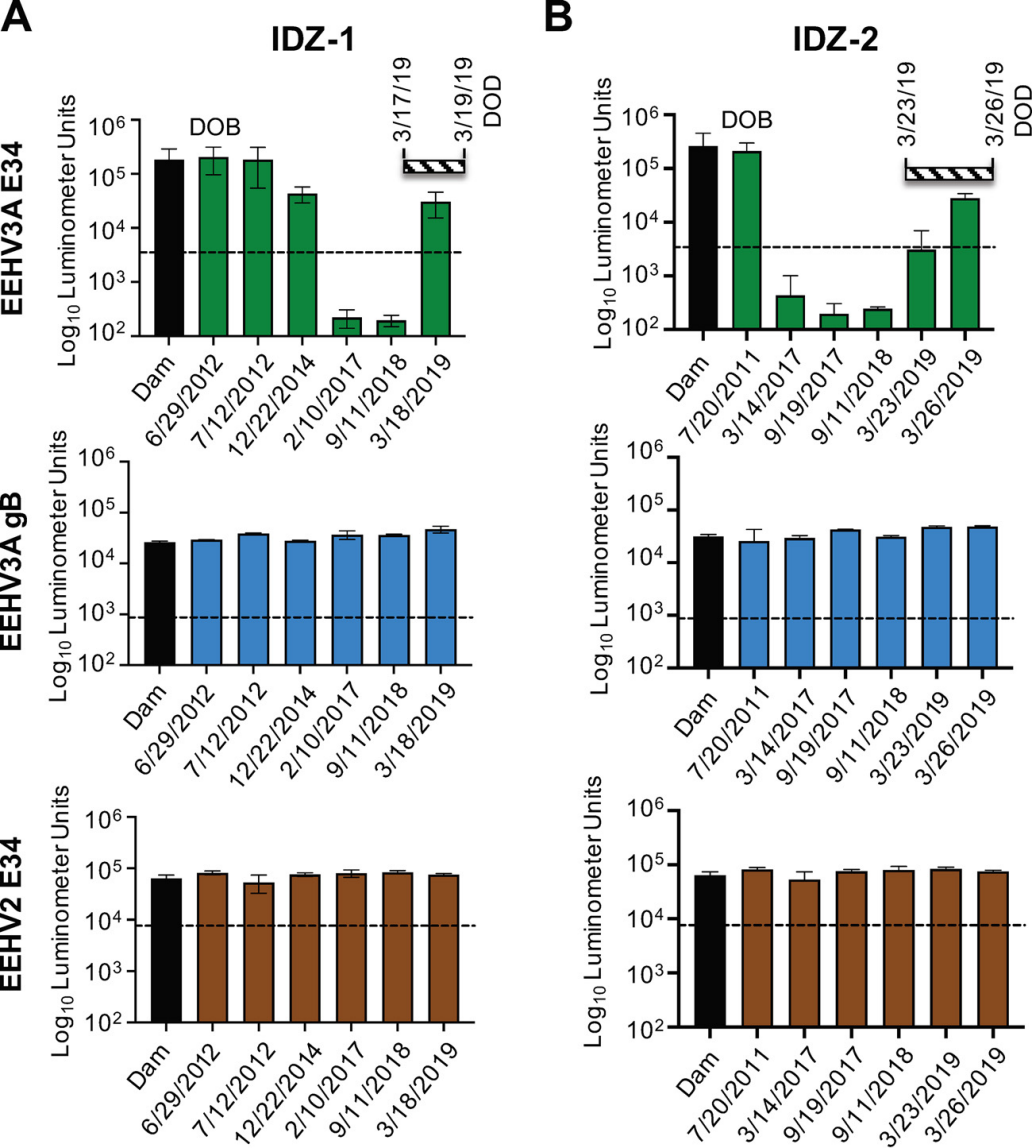
Anti-EEHV3 maternal antibody levels over time in African elephant calves that experienced lethal EEHV-HD. (A) Anti-EEHV antibody levels from birth until the date of death from EEHV-HD for elephant IDZ-1. The specific EEHV antigen tested is indicated to the left of the graphs. The black bar in each graph represents antibody levels detected in the dam on the day of parturition. The striped horizontal bar above the graphs indicates the start date and duration of clinical illness, as well as the date of death (DOD). Antibody levels are expressed in RLUs and plotted on a log_10_ scale. The dashed lines indicate the cutoff levels for determining the sensitivity and specificity for each viral antigen, which were derived from the mean antibody titers of seronegative serum samples or no-serum controls plus 5 SDs. (B) Same as described for panel A for elephant IDZ-2.

**FIG 4:**
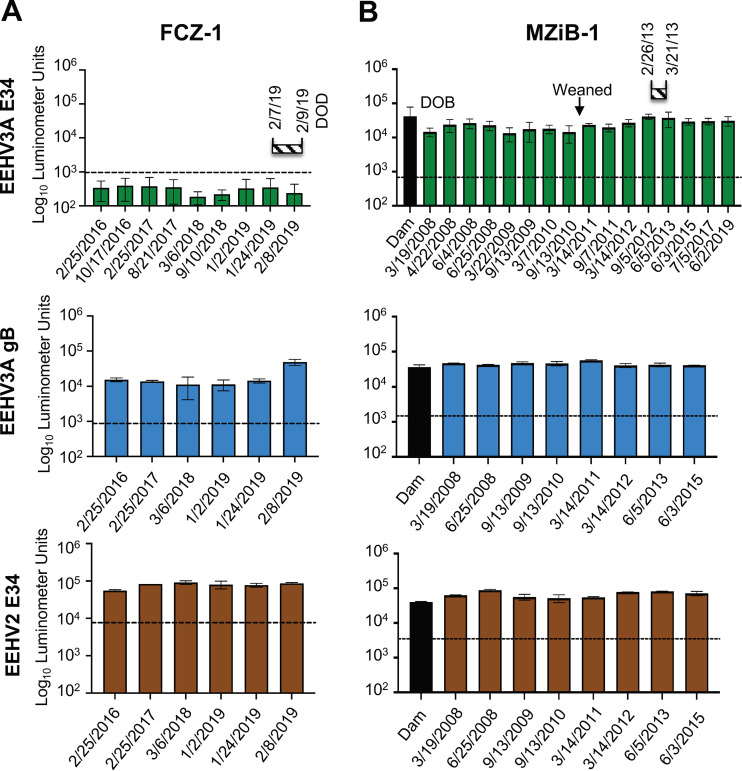
Comparative levels of anti-EEHV3 antibodies over time in African elephant calves. (A) Anti-EEHV antibodies from 2016 until the date of death in 2019 from EEHV-HD for elephant FCZ-1. The specific EEHV antigen tested is indicated to the left of the graphs. The striped horizontal bar above the graphs indicates the start date and duration of clinical illness and the date of death (DOD). Antibody levels are expressed in RLUs and plotted on a log_10_ scale. The dashed lines indicate the cutoff levels for determining the sensitivity and specificity for each viral antigen, which were derived from the mean antibody titers of seronegative serum samples or no-serum controls plus 5 SDs. (B) Same as described for panel A for elephant MZI-1 except that the black bar in each graph represents antibody levels detected in the dam on the day of parturition. Because elephant MZI-1 survived EEHV-HD, the striped horizontal bar above the graphs indicates the duration of clinical illness only. The approximate time when the calf weaned is indicated.

We are unaware of any previous studies investigating the maternal-to-fetal anti-EEHV antibody transfer in African elephants. However, studies in Asian elephants suggest that the majority of antibody transfer occurs prenatally and includes anti-EEHV antibodies (5, 21). Having established and validated serological assays for pan-EEHV and EEHV3-specific antibodies, we tested whether African elephant calves received anti-EEHV3 antibodies from their dams and whether these titers were sustained. We found that in three calves, i.e., IDZ-1, IDZ-2, and MZiB-1, anti-EEHV3A E34 antibody levels at birth were similar or equal to those found in their dams (Fig. 3A and B and Fig. 4B). Available sequential samples from IDZ-1 and IDZ-2 showed decreases in anti-EEHV3 antibody titers, which were undetectable at approximately 5 to 6 years of age. In both IDZ-1 and IDZ-2 calves, anti-EEHV3 E34 titers remained at undetectable levels for more than 1.5 years prior to the development of lethal EEHV3-HD (Fig. 3A and B). However, during their EEHV3 illness and immediately prior to death, anti-EEHV3 E34 titers began to increase, indicating EEHV3 infection and seroconversion. In contrast, these elephants apparently maintained antibody levels from birth toward the pan-EEHV marker gB and the EEHV2 E34 protein (Fig. 3A and B). These results suggest that African elephant calves can receive EEHV-specific antibodies transplacentally and type-specific titers can decline to undetectable levels over time, presumably due to the absence of exposure to one or more of the EEHVs endemic within that herd. Elephant FCZ-1 also had undetectable levels of anti-EEHV3 antibodies for an extended period prior to lethal infection with EEHV3B (Fig. 4A), However, we were unable to assess her anti-EEHV3 serostatus at birth or that of her dam, because those samples are unavailable. FCZ-1 did maintain immunoreactivity to the pan-EEHV gB and EEHV2 E34 proteins over a period of several years, indicating that she had been infected previously with one or more EEHVs other than EEHV3 (Fig. 4A). In contrast, calf MZiB-1 sustained anti-EEHV3A E34 titers from birth until 12 years of age, suggesting that this calf might have acquired EEHV3 infection during a period when his maternal anti-EEHV3 antibody levels were high (Fig. 4B). Interestingly, this elephant developed clinical illness from EEHV3B in February/March 2013 (3). Because we are unable to distinguish between prior infections with EEHV3A or EEHV3B at this time, it is unclear whether this elephant was previously infected with EEHV3A. Alternatively, and in contrast to current evidence in Asian elephants, a possibility remains that some cases of EEHV3-associated disease in African elephants may be caused by reactivation, as opposed to primary infection.

## DISCUSSION

The results presented here provide compelling evidence that primary infection with EEHV3 can underlie fatal EEHV-HD in African elephants (Fig. 1, 3, and 4). We also provide evidence that transplacental transfer of anti-EEHV antibodies occurs in African elephants, similar to Asian elephants (Fig. 3). In addition, maternal antibodies can wane over time to undetectable levels, leaving calves vulnerable to primary infection that may cause illness or death (Fig. 3). Application of the EEHV3 E34 LIPS assay may afford a useful tool for assessing the immune status of African elephants at risk to develop fatal EEHV3 infection.

The EEHV-specific LIPS assay has several key features that support its use in investigating EEHV in African elephants. These features include the following: (i) only small amounts of serum are needed for testing, (ii) species-specific reagents are not required, and (iii) it provides the ability to rapidly produce and screen new antigens. Thus, the LIPS assay system provides a flexible platform to monitor EEHV infection status and to explore EEHV biology in African elephants. The adaptability of LIPS was demonstrated when we identified the E34 antigen, which is both highly immunogenic in elephants and divergent between EEHV species. By incorporating E34 into the LIPS assay, we were able to distinguish between infection with EEHV3 and the other EEHV strains (i.e., EEHV2 and EEHV6) in African elephants. The ability to differentiate between strains is critical to address the knowledge gaps regarding EEHV in African elephants, as well as to determine whether previous cases of EEHV infection were due to primary infection or reactivation.

In this study to characterize the specific seroprevalence of EEHV in African elephants, we found that 100% of African elephants tested had evidence of prior EEHV infection. One limitation of this research is the small number of adult and juvenile animals tested (*n *= 28). Although the assays showed 100% sensitivity and specificity, we anticipate that surveillance of larger numbers of animals will reveal a range of antibody responses in elephants with evidence of chronic infection. Whether this will alter the cutoff value for our EEHV3 E34 serological assay remains to be determined. Our study is also the first to document transplacentally acquired anti-EEHV antibodies in African elephants. Although our study provides evidence for the acquisition and disappearance of maternally transferred EEHV antibodies in some individuals across elephant species, additional juvenile animals will need to be surveyed to determine whether this is broadly representative of most elephants. It seems fairly clear that being seronegative toward a specific EEHV type renders young elephants from both elephant species susceptible to severe primary EEHV infections, leading to death.

While we observed that fatal cases of EEHV3-HD occurred in EEHV3-seronegative animals (i.e., for both EEHV3A and EEHV3B subspecies), two of those elephants began to show seroconversion for E34 during their clinical illnesses with EEHV3-HD (Fig. 3). However, by the time they developed significant anti-EEHV3 E34 titers, it appears that the adaptive immune response was insufficient to protect them from fatal disease. Conversely, survivors of EEHV3-HD were seropositive for at least one EEHV3 subspecies. This was observed in three independent herds, with one outbreak associated with EEHV3A (IDZ-1, IDZ-2, and IDZ-3) and the others associated with EEHV3B (FCZ-1 and MZiB-1). EEHV3A and EEHV3B genomes diverge by approximately 9% on average (15) (G. S. Hayward and P. D. Ling, unpublished data), compared to EEHV1A and EEHV1B genomes, which diverge by approximately 4.5%, with over one-half of ORFs showing less than 1% divergence (1, 8, 13, 14). One explanation for these cases is that the elephants had been infected with one species (i.e., EEHV3A or EEHV3B) prior to being infected with the other and the first infection provided some level of cross-protective immunity. Because no discriminatory serological test exists to differentiate between infections with EEHV3A versus EEHV3B and records of EEHV viremia and shedding history are incomplete, we cannot rule out the possibility that animals suffering nonlethal EEHV-HD from one EEHV3 subspecies were experiencing a reactivation event. This seems less likely, however, given the evidence in Asian elephants, which suggests that both EEHV-related morbidity and death are the result of primary infections (5).

Use of the LIPS assay to identify potentially EEHV3-seronegative African elephants, which we now show may be associated with the development of lethal EEHV-HD cases in African elephants, could be an important tool in elephant management. Serostatus can be taken into account when assessing the risk of transferring elephants between facilities. Additionally, in cases in which low-level viremia is detected, serostatus and other clinical data can be used to determine whether to initiate treatment for potential EEHV disease. These treatments often involve a number of disruptive, costly, and stressful activities for both zoo personnel and the elephant herd. Lastly, this assay will be a necessary tool to evaluate the efficacy of future candidate vaccines against EEHV.

## MATERIALS AND METHODS

### Study sera.

Serum samples were obtained from 11 African elephants at the San Diego Zoo Safari Park (SDZSP). At least 5 of the elephants had evidence of latent EEHV infection from either an EEHV-positive trunk wash or viremia (L. Howard and E. Latimer, unpublished data) (Table 1). Sera were also obtained from three other institutions where there was a clinical or fatal EEHV-HD case associated with EEHV3 (6); these included 8 elephants housed at the Indianapolis Zoo (IDZ), 5 from the Fresno Chaffe Zoo (FCZ), and 4 from the Maryland Zoo in Baltimore (MZiB) (Tables 2 and 4).

### Determination of novel EEHV protein sequences and preparation of luciferase-antigen fusion constructs.

The EEHV3A U39 and E34 proteins were generated from genomic sequencing of EEHV3A Nyah (accession number MN373268). Similarly, the genomic sequences for EEHV3B, EEHV2, and EEHV6 were determined (G. S. Hayward and P. D. Ling, unpublished data). The U39 sequences for EEHV2 and EEHV6 were described previously (13, 14). Codon-optimized sequences for predicted EEHV U39 and E34 proteins were synthesized (Genscript) and cloned in frame with the GLuc gene in the pGaus3 expression plasmid (5, 22, 23). Generation of fusion proteins was performed as described previously (5).

### LIPS analysis.

LIPS analysis was performed as described previously (5, 24). Briefly, serum samples were initially diluted 1:10 in buffer A (50 mM Tris [pH 7.5], 100 mM NaCl, 5 mM MgCl_2,_ 1% Triton X-100) for storage up to 1 month at 4°C. For use in the assay, samples were diluted 1:5 in the same buffer. Luminescence units (LU) of stored GLuc extracts were determined on the day of the assay, and a master mix of each GLuc extract containing 1 × 10^7^ LU per 50 μl was made. Fifty microliters of GLuc extract master mix was added to 50 μl of diluted serum (in duplicate) and incubated for 1 h at room temperature on a shaker. After 1 h, GLuc extract-diluted serum was added to 5 μl of a 30% protein A/G-bead suspension (diluted in phosphate-buffered saline [PBS]) in a 96-well filter plate (Millipore). Samples were incubated with the beads for 1 h at room temperature on a shaker. Beads were then washed five times with buffer A, followed by two PBS washes, using a 96-well vacuum manifold (Millipore). An OmniStar luminometer (BMG Labtech) was used to inject 50 μl of *Renilla* luciferase assay substrate into each well, shake the plate for 2 s, and then record relative light unit (RLU) values for 5 s. Values reported represent the average RLUs over 5 s postinjection.

### Immunoblot assays.

For analyses of EEHV *Gaussia* fusion proteins, supernatants or extracts from cell-associated GLuc fusions were mixed with 2× sample loading buffer and resolved by SDS-PAGE, and immunoblotting was carried out as described previously (5). A commercially available rabbit anti-*Gaussia* polyclonal antibody (New England Biolabs) was used to detect the EEHV-GLuc fusion proteins.

### Statistical analysis.

Statistical analysis was performed with GraphPad Prism software, version 8.4.3. In general, LIPS assays were performed in duplicate, with at least two independent experiments for each sample. Results for different treatment groups are presented as geometric means ± standard deviations (SDs). Group means were compared with Mann-Whitney *U* tests, and significance was set at *P* values of ≤0.05. The cutoff value for determining sensitivity and specificity for each viral antigen was derived from the mean antibody titer of the uninfected samples plus 5 SDs.

### Data availability.

The E34 sequences derived for EEHV3B, EEHV2, and EEHV6 were deposited in GenBank with accession numbers MZ269261, MZ269260, and MZ269262, respectively.
